# Predictive Validity of the Suicide Trigger Scale (STS-3) for Post-Discharge Suicide Attempt in High-Risk Psychiatric Inpatients

**DOI:** 10.1371/journal.pone.0086768

**Published:** 2014-01-21

**Authors:** Zimri S. Yaseen, Irina Kopeykina, Zinoviy Gutkovich, Anahita Bassirnia, Lisa J. Cohen, Igor I. Galynker

**Affiliations:** 1 Beth Israel Medical Center, New York, New York, United States of America; 2 St. Luke's Roosevelt Hospital, New York, New York, United States of America; Chiba University Center for Forensic Mental Health, Japan

## Abstract

**Background:**

The greatly increased risk of suicide after psychiatric hospitalization is a critical problem, yet we are unable to identify individuals who would attempt suicide upon discharge. The Suicide Trigger Scale v.3 (STS-3), was designed to measure the construct of an affective ‘suicide trigger state’ hypothesized to precede a suicide attempt (SA). This study aims to test the predictive validity of the STS-3 for post-discharge SA on a high-risk psychiatric-inpatient sample.

**Methods:**

The STS-3, and a psychological test battery measuring suicidality, mood, impulsivity, trauma history, and attachment style were administered to 161 adult psychiatric patients hospitalized following suicidal ideation (SI) or SA. Receiver Operator Characteristic and logistic regression analyses were used to assess prediction of SA in the 6-month period following discharge from hospitalization.

**Results:**

STS-3 scores for the patients who made post-discharge SA followed a bimodal distribution skewed to high and low scores, thus a distance from median transform was applied to the scores. The transformed score was a significant predictor of post-discharge SA (AUC 0.731), and a subset of six STS-3 scale items was identified that produced improved prediction of post-discharge SA (AUC 0.814). Scores on C-SSRS and BSS were not predictive. Patients with ultra-high (90^th^ percentile) STS-3 scores differed significantly from ultra-low (10^th^ percentile) scorers on measures of affective intensity, depression, impulsiveness, abuse history, and attachment security.

**Conclusion:**

STS-3 transformed scores at admission to the psychiatric hospital predict suicide attempts following discharge among the high-risk group of suicidal inpatients. Patients with high transformed scores appear to comprise two clinically distinct groups; an impulsive, affectively intense, fearfully attached group with high raw STS-3 scores and a low-impulsivity, low affect and low trauma-reporting group with low raw STS-3 scores. These groups may correspond to low-plan and planned suicide attempts, respectively, but this remains to be established by future research.

## Introduction

The increased risk of suicide in the period after discharge from a psychiatric hospital is a critical problem, yet we lack the ability to evaluate post-discharge suicide risk in a clinically meaningful way. As many as 6% of all suicides by discharged patients occur in the first week after discharge [1] and 20–25% occur within the first 3–12 months [2], [3]. Further, the adjusted risk ratio for completed suicide in the first week after discharge, compared to that of never hospitalized stands at a startling 102∶1 for men and 246∶1 for women [4]. Thus while the period following psychiatric hospital discharge is crucial for suicide prevention efforts, no factor or combination of factors used to assess long-term suicide risk are of clinical value in predicting short term suicide risks after discharge from the hospital, as 60% of patients who commit suicide are categorized as low risk [3].

Most work on suicide risk assessment has focused on *chronic* traits and risk factors. Thus, despite considerable and wide-ranging efforts, risk-factor based approaches, while of significant value at an actuarial and public health level, have never fully achieved clinical significance [5], [6]. However, a small but growing body of evidence characterizes a suicide crisis as an *acute state* which can precipitate the transition from chronic SI to acute SA [7]–[9]. Further, this state appears to have relations to certain forms of panic [10]–[12]. Consistent with the acute state hypothesis of transition from SI to SA, a study by Deisenhammer et al., [13] reported that the transition from the first emergence of suicidal ideation to actual suicide attempts is typically as short as ten minutes. Thus, in our previous work on suicide we have sought to describe a distinct panic-like syndrome, hypothesized as a “suicide trigger state” combining frantic hopelessness, ruminative flooding, and near-psychotic somatization. In these studies we developed a self-report measure, the ‘Suicide Trigger Scale’ (STS) to assess this “suicide trigger state” and evaluated its relation to suicide attempts and ideation [14], [15]. In the first such study, we explored the factor structure of an earlier version of the scale, the STS-2, and found that higher scores associated with a past history of suicide attempt among psychiatric inpatients [14]. In a follow-up study the STS-2 was modified to its current form, the STS version 3 (STS-3), and its structure reanalyzed on a larger sample of suicidal patients presenting in the psychiatric emergency room setting. The results showed a correlation between the total score on the STS-3 and severity of suicidal ideation, as well as an association between higher “frantic hopelessness” scores and presence of an actual suicide attempt at the time of presentation to the Emergency Room (ER) [15]. While these studies demonstrated excellent construct validity with regard to past suicidal behavior, *predictive* validity for the STS has not yet been demonstrated. In this light, the present study aims to test the predictive validity of the STS-3 for post-discharge suicide attempt on the high-risk sample of psychiatric patients admitted to an inpatient unit with suicidal ideation or attempts.

## Methods

The 42-item STS-3 (henceforth referred to as the STS) and a psychological test battery including measures of suicidality, depression, affective intensity, impulsivity, and attachment style were administered following admission to 175 adult psychiatric patients hospitalized following suicidal ideation or attempt to the psychiatric unit. Study participants were also assessed 2–6 months after discharge for post-discharge suicidality. The study was approved by the Beth Israel Medical Center Institutional Review Board.

### Study Setting

Participants were recruited from the inpatient psychiatric units of Beth Israel Medical Center (BIMC) and St. Luke’s Roosevelt Hospitals (SLR) in New York City from December, 2010 through October, 2012. Beth Israel is a 1,368-bed, full-service community and tertiary care teaching hospital in Manhattan's Lower East Side. The inpatient psychiatric service maintains a 92-bed capacity. St. Luke’s-Roosevelt Hospital is a 1076-bed, full-service community and tertiary care teaching hospital on Manhattan's Upper West Side. The inpatient psychiatric service maintains a 69-bed capacity. These hospitals serve similar diverse urban populations.

### Inclusion and Exclusion Criteria

Included were males and females ages 18–65, admitted to an inpatient unit for psychiatric hospitalization after presenting to the ER with suicidal ideation or attempt as documented in their patient chart. Thus the study population consisted of adult psychiatric inpatients with suicidal ideation or attempt (as defined by the Columbia Suicide-Severity Rating Scale (C-SSRS) [16]) which led to their admission. Patients included had to be able to understand the nature and substance of the informed consent. Potential participants were excluded in case of homelessness (due to difficulty of follow-up), or if mental retardation, cognitive impairment, or linguistic limitation precluded understanding the consent or research questions, or if significant medical or neurological disease or possible delirium which might interfere with participation was present. 175 patients consented to participate and 157 patients either declined (n = 84) or were ineligible (n = 73) to participate. Of the 73 that were ineligible, 32 did not meet age inclusion criteria, 36 were unable to provide informed consent, 3 received a diagnosis of malingering, 1 was homeless, and 1 had unspecified ineligibility.

### Informed Consent

Potential participants were identified and referred to the study by their inpatient clinicians on the psychiatric inpatient unit to which they had been admitted. Trained research assistants then approached each identified potential participant to explain the study, its aims, and risks and benefits of participation. In order to avoid reporting bias that might be associated with presenting the study as explicitly focused on suicidality, the consent form was altered. The title of the study was changed to “Predicting Emotional Dysregulation: Internal Consistency and Predictive Validity of the Emotional Dysregulation Scale” and the phrases “suicidal ideation” and “suicide attempts” were replaced with the term “emotional dysregulation.” If the patient agreed to participate, he or she was then presented with a “Health Insurance Portability and Accountability Act” (HIPAA)-compliant consent form to review and sign. The informed consent included permission to review the patient’s medical record on the unit, and to contact the patient in the future for follow-up assessment.

### Initial Assessment

Participants were administered a packet that included the STS [15] and two measures of related constructs and symptomatology, the Columbia Suicide-Severity Rating Scale (C-SSRS) [16] and Beck Scale for Suicidal Ideation (BSS) [17], [18]. Measures of associated symptom domains included the Beck Depression Inventory (BDI) [19], [20], the Affective Intensity Rating Scale (AIRS) [21], and the Barratt Impulsivity Scale (BIS-11) [22]. The Brief Symptom Inventory (BSI) [23] was included as a general measure of symptomatology. The Relationship Style Questionnaire (RSQ) [24], [25]; the Parental Bonding Instrument (PBI) [26]; the Measure of Parental Style (MOPS) [27]; and the Childhood Trauma Questionnaire (CTQ) [28] were included as measures of attachment style and developmental experiences, and the Suicide Opinion Questionnaire (SOQ) [29] provided a broad survey of attitudes and opinions regarding suicide in general.

Finally, psychiatrist-determined clinical diagnoses for study participants were gathered from patient charts’ discharge summaries. Psychiatrists were experienced board certified staff psychiatrists or psychiatry residents directly supervised by the former. Axis I diagnoses were then coded as 1) No primary DSM-IV Axis I mood, anxiety, or psychotic disorder (this category comprised primary diagnoses of Borderline personality d/o, adjustment and substance induced disorders, primary diagnoses of substance dependence, and diagnoses recorded as “Mood disorder not otherwise specified”), 2) Anxiety or unipolar depressive disorders, 3) Bipolar I or II disorders, and 4) Psychotic disorders. Diagnoses were condensed into four categories to maximize degrees of freedom, (thereby increasing statistical power in subsequent analyses) as well as diagnostic reliability [30]–[32].

### Follow-up Assessment

Starting at two months post-discharge, attempts were made to contact participants for follow-up assessment of suicidality. Participants who could not be reached within six months of discharge or who refused to participate were considered lost to follow-up. The primary endpoint assessed was presence or absence of a suicide attempt during the post-discharge follow-up period. The C-SSRS was used to assess the presence of post-discharge suicide attempts, defined as ‘potentially self-injurious acts committed with at least some wish to die, as a result of those acts’ [33]. In addition, the US national death registry and patient medical records at the participating hospitals were searched for any deaths and ER visits or hospitalizations for suicide attempt (as defined above) following discharge.

### Statistical Analysis

To test the predictive validity of the STS for post-discharge suicide attempt we used Receiver Operator Characteristic (ROC) area under the curve (AUC) and optimal cut-point analyses in addition to univariate and multivariate binary logistic regressions with suicide attempt during the 6-month post-discharge follow-up period as the outcome variable. Discriminant function analyses were performed to identify any individual STS items discriminating between post-discharge attempters and non-attempters. Lastly, to assess the relationship between the subscale and the complete STS, exploratory principal axis factor analysis with varimax rotation was used to assess the subscale structure, and subscale score was correlated with STS total score. Seven participants who otherwise provided adequate data for inclusion in the study sample failed to respond to one of the 42 items on the STS (a different item was skipped by each participant). Missing data on an item was scored as 1 (approximately the aggregate mean item response for all items and all participants).

## Results

### Sample Demographics

Of the 175 participants recruited, 161 provided adequate data for inclusion in the study sample. Of those, follow-up data was obtained for 54 participants (33%), while the rest were lost to or refused follow-up: 12 were reached but refused follow-up, 7 were scheduled but did not arrive for follow-up, and 20 had wrong or out of service phone-numbers, and 68 were unreachable by phone, e-mail, or letter following 4 phone calls, 2 letters, and 2 emails within 6 months of discharge. None were listed in the US death registry.

Participants followed up did not differ significantly (p>0.08) from those lost to follow-up in terms of age, sex, level of education, marital status, income bracket, homelessness, rates of history of incarceration, substance use, presence of suicide attempt leading to admission, diagnosis, or STS total scores. See Table 1a&b.

**Table 1:** 

Table 1a – Behavioral and Demographic Characteristics of Study Sample						
	Followed Up?					
	No		Yes		Total	
	N Reporting	Mean[SD]/%	N Reporting	Mean[SD]/%	N Reporting	Mean[SD]/%
Initial Suicide Attempt*	108	36%	54	43%	161	38%
Substance or Alcohol Use	107	61%	54	56%	161	59%
Age (years)	106	37.04 [12.9]	54	39.17 [13.0]	160	37.76 [13.0]
Male	106	44%	54	52%	160	47%
Married	106	12%	51	4%	157	10%
Years of Education	89	13.22 [3.0]	44	12.82 [3.2]	133	13.09 [3.1]
Income bracket (rank)	105	1.63 [1.3]	53	1.62 [1.3]	158	1.63 [1.3]
Domiciled	106	88%	51	88%	157	88%
History of Incarceration	103	31%	53	30%	156	31%
STS Total	102	49.07 [18.2]	51	49.78 [19.6]	153	49.31 [18.6]
**Table 1b** ** – Clinical Characteristics of Study Sample**						
	**Followed Up?**					
	**No** **		**Yes**		**Total**	
**Discharge Diagnosis Category**	**Count**	**% of Subtotal**	**Count**	**% of Subtotal**	**Count**	**% of Subtotal**
Primary psychotic d/o	16	15.2%	11	20.4%	27	17.0
Primary bipolar mood d/o	16	15.2%	6	11.1%	22	13.8%
Primary unipolar mood or anxiety d/o	39	37.1%	22	40.7%	61	38.4%
No Primary mood, anxiety, or psychotic d/o	34	32.4%	15	27.8%	49	30.8%

* Indicates suicide attempt leading to hospital admission. SD = Standard Deviation.

** 2 subjects lost to follow up did not have recorded diagnoses.

Of the 54 participants with follow-up data, 13 (24.1%) reported a post-discharge suicide attempt, which was within the expected range of 20–25% [34], [35].

### STS Distribution and STS Score Transformation

**Figure 1:**
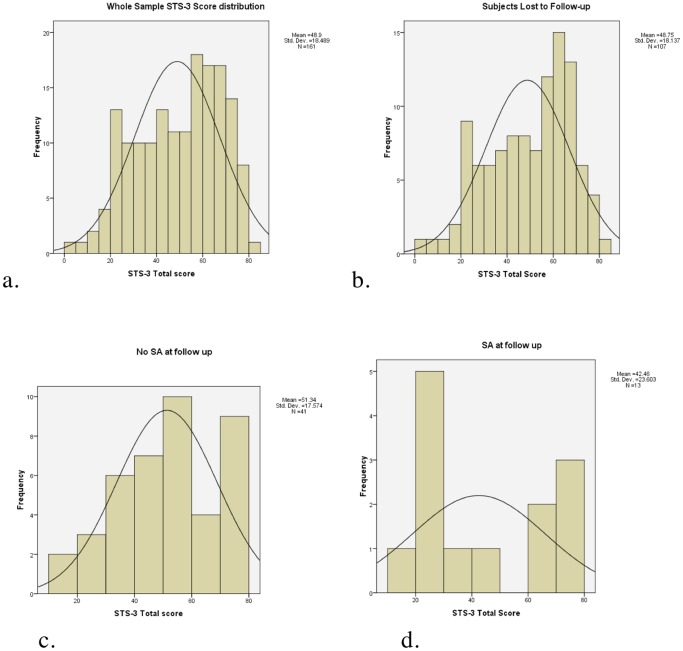
STS score distributions for a. entire sample, b. subjects lost to follow-up, c. subjects without post discharge suicide attempt, and d. subjects with post-discharge suicide attempt.

When examined for normality and skewness, the distribution of STS scores for entire sample (n = 161) was not normal (Kolmogorov-Smirnov statistic 0.106, p<0.0005), with skewness of −0.4 indicating a moderate left skewing of the distribution). STS score distributions of participants without post-discharge attempt and those lost to follow-up, were similar to those from the sample as a whole. (See figure 1a–c.).

The distribution of STS scores for participants with post-discharge SA, however, was bimodal and differed markedly from the previous three distributions (Figure 1d). For this group 15% participants scored between 30 and 60 on the STS (Sarle’s bimodality coefficient = 0.83), whereas for participants without post-discharge SA, 55% of participants scored between 30 and 60 (Sarle’s bimodality coefficient = 0.54). [36].

Given the bimodality of the SA score distribution the STS scores were transformed to distinguish high and low scores from intermediate scores. Because of the left skewness of the entire sample scores, the transformed score was calculated as the absolute value of the total score minus the median score, rather than the mean score.

### STS Transformed Score Predictive Validity

**Figure 2:**
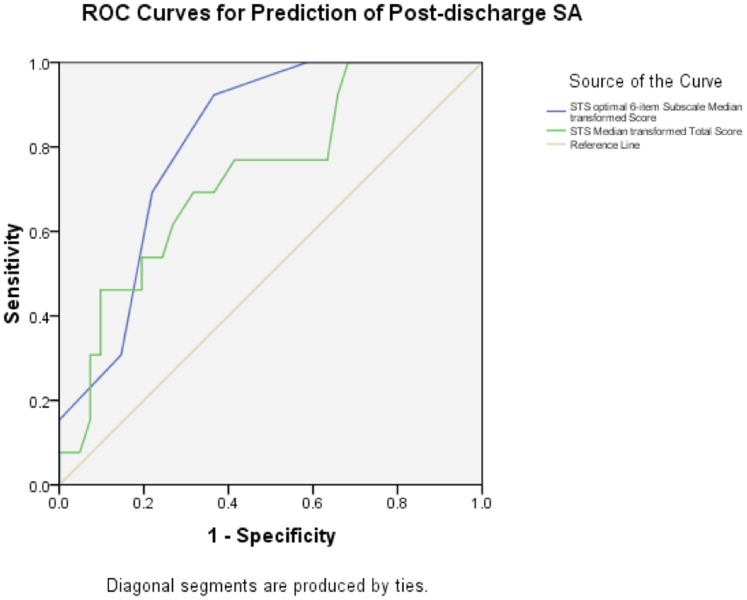
ROC curves for prediction of post-discharge SA by median transformed total (AUC = 0.731) and optimal 6-item subscale (AUC = 0.814) scores.

Although the STS raw scores did not distinguish SA and non-SA groups, the transformed score significantly differed between suicide attempters and non-attempters during the follow-up period (Mann-Whitney U 2-tailed p = 0.015). ROC analysis of the transformed score was significant (AUC = 0.731, p = 0.013) with sensitivity of 0.692 and specificity of 0.683 for post-discharge SA at the optimal cut score of ≥19. (See figure 2.) Using this cut score, 37 of 54 (68.5%) participants were correctly classified. Positive predictive value was 40.9% and negative predictive value 87.5%.

In univariate binary logistic regression the odds ratio (OR) for transformed STS score ≥19 was 4.85 (p = 0.022). When SA at admission, age, gender, substance use and diagnostic category were included in the model the relationship remained significant with AOR = 5.41 (p = 0.033). AORs for the other variables did not approach statistical significance in the model.

Of note, BSI global severity index, BDI, BSS part 1, part 2, and total scores, AIRS, BIS total and subscale scores, RSQ subscale, and CTQ subscale and total scores were not significant predictors of SA in binary logistic regression analyses. Of CSSR-S measures of suicidality at intake (SI type, duration, frequency, controllability, presence of preparatory acts, past suicide history and self-reported number of attempts), no measure at admission was a significant predictor of post-discharge SA.

Finally, in one-at-a-time binary regression analyses of socio-demographic and basic clinical factors (age, sex, income, marital status, homelessness, history of incarceration, substance use, diagnostic category, presence of a suicide attempt at admission, and length of stay), none were significant predictors of SA.

### Exploratory Analyses of Ultra-High versus Ultra-Low Scorers on the STS

Given that unusually high and unusually low scores on the STS during hospitalization were both associated with post-discharge SA, we defined ultra-low (UL) scorers as those in the bottom 10% (scoring 22 or less) and ultra-high (UH) scorers as those in the top 10% (scoring 72 or more). The UL and UH cohorts thus represented samples of patients who, respectively, either denied or strongly endorsed almost all symptoms in the STS. We then sought to compare these groups on the range of demographic and associated clinical constructs we assessed to determine if UH and UL scorers on the STS represented distinct patient types.

UL, intermediate, and UH scoring post-discharge suicide attempters (n = 13) were compared in terms of affective intensity (AIRS total score), depression (BDI total score), multiple domains of impulsiveness (BIS subscale scores), abuse history (CTQ subscale scores), and select attachment domains (RSQ subscale scores). Emotional abuse scores were significantly higher for UH than UL scores (p = 0.017). When the STS group analysis for UL vs. UH scorers was expanded to the entire sample to improve statistical power, for the UH vs. UL scorers, the differences in affective intensity, fearful attachment, physical abuse, and non-planning impulsiveness all reached statistical significance (ANOVA F-tests with criterion alpha = 0.05, Bonferroni corrected criterion alpha = 0.0025). Group differences in AIRS and BDI were also statistically significant, while BSS and CSSR-S frequency and severity of ideation were not (ANOVA F-test p>0.05, (See Table 2).

**Table 2:** Clinical Characteristics of STS score-groups: ANOVA Comparison of Means.

	STS Score Group							
	Ultra-Low		Intermediate		Ultra-High			
	NReporting	Mean[Std. Deviation]	NReporting	Mean[Std. Deviation]	NReporting	Mean[Std. Deviation]	BetweenGroups p	Linearity p
AIRS	17	7.76 [4.777]	121	14.91 [5.670]	18	20.89 [4.418]	<0.0005	<0.0005
BDI	14	14.93 [10.381]	116	25.93 [11.489]	18	31.17 [12.297]	<0.0005	<0.0005
BIS Attentional Impulsiveness	8	14.50 [3.388]	65	18.31 [4.700]	13	22.77 [3.609]	<0.0005	<0.0005
BIS Motor Impulsiveness	7	19.14 [6.866]	66	24.09 [6.149]	13	29.23 [6.906]	0.003	0.001
BIS Non-Planning Impulsiveness	7	20.29 [5.707]	69	28.10 [5.286]	13	31.69 [5.865]	<0.0005	<0.0005
BSS	9	12.56 [9.888]	92	16.90 [8.828]	17	19.00 [7.850]	0.209	0.093
CTQ Emotional Abuse	15	11.33 [7.228]	120	13.07 [5.819]	18	18.56 [5.596]	0.001	<0.0005
CTQ Physical Abuse	16	10.19 [6.775]	119	9.98 [5.299]	18	17.94 [5.631]	<0.0005	<0.0005
CTQ Sexual Abuse	15	9.20 [6.868]	119	8.90 [6.210]	18	14.78 [8.186]	0.002	0.009
RSQ Secure Attachment	16	16.25 [3.697]	123	14.01 [3.437]	17	13.00 [2.784]	0.018	0.007
RSQ Preoccupied Attachment	16	12.25 [4.740]	118	13.38 [3.258]	17	13.53 [2.695]	0.434	0.288
RSQ Dismissing Attachment	16	18.00 [3.578]	120	17.65 [3.429]	17	18.06 [3.631]	0.855	0.949
RSQ Fearful Attachment	15	9.40 [3.757]	123	13.46 [3.953]	17	15.12 [4.256]	<0.0005	<0.0005

Finally, there was no statistical difference between UL and UH scorers in terms of rates of follow-up, homelessness, history of incarceration, married status, or male vs. female sex (using 2-tailed Pearson’s Chi-squared and Fisher’s exact tests with criterion alpha = 0.05) or in terms of age, years of education completed, or annual income (ANOVA F-tests with criterion alpha = 0.05).

### STS Predictive Subscale Identification and Analysis

In an exploratory secondary analysis, we sought to identify a predictive subscale of the STS-3 without over-fitting the data. To capture signal from the clinically distinct high- and low-scorers while maintaining statistical power, the sample was divided into lower (STS total<41) and upper tertile (STS total >59) scoring cohorts; separate linear discriminant function analyses were performed on lower and upper-tertile scoring participants to identify any individual STS items discriminating (p<0.05) between post-discharge attempters and non-attempters for each of these groups. This analysis was repeated to determine items discriminating between subjects with SA at admission (n = 62) vs. SI only (n = 99) among the separate high- and low-scoring subsamples to provide greater power for item identification. The above analyses were also performed across the entire sample. Finally, the distance from median transform was applied to each individual STS item and transformed STS items were then compared across post-discharge attempters and non-attempters and between subjects with SA vs. SI only at admission in all subjects.

Only items that were significant in at least two of the analyses were included in the subscale in order to limit false discovery of contributing items. The subscale was then tested as a predictor of post-discharge SA using ROC and binary logistic regression analyses.

Seven individual items were identified as significantly associated with SA in at least two distinct analyses. (See table 3): 2. “Did you feel your thoughts are confused?” 4. “Did you feel there is no exit?” 7. “Did you feel that your head could explode from too many thoughts?” 23. “Did you feel bothered by thoughts that did not make sense?” 27. “Did you feel trapped?” 39. “Did you feel pressure in your head from thinking too much?” and 41. “Did you feel like you were getting a headache from too many thoughts in your head?”.

**Table 3:** Identification of items for STS predictive subscale.

*Group:*	Lower Tertile STS scorers				Upper Tertile STS scorers				All Subjects				All Subjects(Individually Transformed Items)			
*Outcome measure:*	SA at admit		SA post discharge		SA at admit		SA post discharge		SA at admit		SA post discharge		SA at admit		SA post discharge	
*Analysis: (STS item # below):*	T-test	Discrim Fxn	T-test	Discrim Fxn	T-test	Discrim Fxn	T-test	Discrim Fxn	T-test	Discrim Fxn	T-test	Discrim Fxn	T-test	Discrim Fxn	T-test	Discrim Fxn
2	x								x							x
4	x			x												
7			x												x	
23			x	x											x	x
27	x	x					x	x			x	x	x	x		
39			x		x	x										
41	x								x	x					x	

Cells marked with an ‘x’ indicate analyses (column) for which a given item on the STS (row) was significant with p<0.05.

To test the contribution of each item to the subscale, the items were removed one at a time and the resulting AUC assessed. The AUC for prediction of post-discharge SA was decreased by exclusion of items 2, 4, 7, 23, and 27, and increased when items 39 and 41 were excluded. When both items were excluded simultaneously however, the AUC was reduced. Thus, an optimal 6-item subscale was identified, comprising items 2,4,7,23,27 and 41, with a median score of 7.

The AUC for the median transformed score was 0.814, p = 0.001 with sensitivity 0.923 and specificity 0.634 at a cut point of score>2, and sensitivity 0.692 and specificity 0.780 at a cut point of score>3. (See figure 2). Furthermore, in multivariate binary logistic regression analyses the 6-item subscale was a significant predictor of post-discharge SA after adjusting for patient age, gender, substance use, attempt at admission, and diagnostic category with AOR of 8.0 (p = 0.009). Age was the only significant covariant in the model with AOR = 0.96 (p = 0.033).

Exploratory principal axis factor analysis with varimax rotation on the 6-item subscale replicated the two primary factors of ‘Frantic Hopelessness’ (comprising items 4 and 27) and ‘Ruminative Flooding’ (comprising all other items) previously found for the STS-3, and accounting for 47.4% of the total variance. All items loaded on these factors as they had in our previously published analysis of the STS-3 with the exception of item 23, which previously had failed to load above threshold on any factor [15]. Scores on this subscale correlated strongly and significantly with total STS-3 score (r = 0.882, p<0.0005).

## Discussion

The primary aim of this study was to test the predictive validity of the STS for post-discharge suicide attempt in a high-risk population – psychiatric inpatients admitted for suicidal ideation or attempt. Our primary finding was that a pattern of extreme responses – substantial denial or very strong endorsement of the STS construct *was* moderately predictive of post-discharge suicide attempts, with sensitivity and specificity of approximately 70% at a threshold of approximately one standard deviation or more from the population mean. Further, the positive predictive value of 41% though modest is high enough to be a potentially useful indicator of a need for modification of treatment plans such as inclusion of an enhanced follow-up [37].

In addition, we found in an exploratory secondary analysis of the data that a brief subscale of the STS replicated the global scale structure and correlated strongly with total scale score while providing superior prediction of post-discharge SA. These results require replication as item selection involved some fitting of the data, but are promising both in terms of the effect size (AOR = 8.0) and clinical utility, as they describe a coherent syndrome within a highly practicable list of six Likert rated questions.

The behavior of the scale merits close attention however, as the relationship between construct endorsement and subsequent suicide attempts is non-linear. Both very high and low scores predicted post discharge SA.

The association of high scores with SA is intuitive, as, in terms of both face and concurrent validity (viz., table 2 linear relations between STS scores and BDI and BSS scores), the scale measures a state of psychic distress most prominently characterized by frantic hopelessness (comprising both a sense of need to escape one’s situation and hopelessness of doing so), and ruminative flooding (which describes a state of being overwhelmed by the volume of negative ruminations). Frantic hopelessness is consistent with the large and intuitive body of evidence linking suicide to hopelessness (e.g., [38], [39]) and the escape theory of suicide [40]. On the other hand, ruminative flooding is suggestive of a state of prefrontal cortex overload, which may expectably result in impaired decision-making [41], [42] and associate bi-directionally with cognitive rigidity [43], which has also been implicated in suicidality [44], [45]. Indeed, behavioral insensitivity to contingency change in a gambling task was recently found to associate with both impulsivity and low-plan (but not low-lethality) suicide attempts among a balanced sample of depressed elderly [46].

The association of scores more than a standard deviation below the population mean with post discharge suicide attempt is a surprising finding, however. Findings detailed in table 2 indicate these individuals are significantly less impulsive, with lower affective intensity, and report less childhood abuse and more secure and less fearful attachment styles. They also report less suicidality and depression. Interestingly (see table 4), self-reports of positive as well as negative affect on the AIRS [21] increase with STS score group. In other words, UL scorers report lower levels of both positive and negative affects than intermediate and UH scorers, who report higher levels of negative but also higher levels of positive affect. These findings in sum suggest that unusually low scores on the STS (given the setting of in-patient hospitalization for suicidality) may be indicative of generalized inhibition of affective communication. While studies of alexithymia have generally failed to find an association between alexithymia and suicidality, *per se* [47], inhibited communication of affect in facial micro-expression analysis has been differentially linked to suicidal behavior among depressed patients [48].

**Table 4:** Affective Intensity of STS Score-groups by Domain: ANOVA Comparison of Means.

	STS Score Group							
	Ultra-Low		Intermediate		Ultra-High		Total	
	N	Mean[Std. Deviation]	N	Mean[Std. Deviation]	N	Mean[Std. Deviation]	N	Mean[Std. Deviation]
Negative Self-Directed Affect*	17	3.24 [2.66]	125	6.86 [2.39]	18	8.61 [1.46]	160	6.67 [2.67]
Positive Self-Directed Affect**	17	1.71 [2.59]	122	3.09 [2.68]	18	4.50 [2.50]	157	3.10 [2.72]

* Between groups & linearity p<0.0005.

** Between groups & linearity p = 0.009, 0.002, respectively.

Taken together, these findings point to the heterogeneity of suicide attempters, and are suggestive to two distinct clinical groups – the first characterized by high impulsivity and affectivity with insecure attachment style, and a second group with low impulsivity and affectivity and a secure attachment style. These groups may in turn correspond to low- and high- plan suicide attempter groups, respectively [49], and could contribute to differences observed in therapist response to suicide attempters [50].

These findings are notable also because they may explain the lack of predictive value derived from other measures of mood and suicidality in this study. Thus, for example, while C-SSRS ratings of severity of SI and SI history are indicative of increasing levels of SA risk among a general psychiatric population [51], among high risk patients these data were uninformative; where suicidality is present implicitly, lack of explicit communication of suicidal ideation may not imply resolution of suicide risk [52], [53].

### Limitations

Two primary limitations of this study must be noted. First, the sample of patients reached for follow-up is small, limiting power to detect effects of potential mediating or moderating variables such as diagnostic category and other demographic characteristics.

Second, positive findings were derived from fitting procedures. Thus, while these procedures were conservative –simple median transforms of STS scores, and exploratory item selection without weighting, independent replication of these findings, particularly of the subscales identified is needed.

Finally, approximately one third of eligible patients declined to participate, and it cannot be determined if such patients differed from included patients in other important respects or would have altered scale performance.

## Conclusions

The relationship between the STS and suicide is complicated, however very high and very low scores at admission to the psychiatric hospital appear to predict suicide-attempts following discharge among the high-risk group of patients admitted for suicidal ideation or attempts. Extreme scorers appear to comprise two clinically distinct groups; an impulsive, affectively intense, fearfully attached group with high levels of childhood trauma who score at last a standard deviation above the mean on the STS, and a low-impulsivity, low affect and trauma reporting group who produce scores at last a standard deviation below the mean on the STS. These groups may correspond to lower- and higher-plan type suicide attempters, respectively, but that remains to be established by future research. Finally, a six-item subscale of the STS-3 demonstrated clinically significant predictive power for post-discharge suicide attempt. However, replication is needed for this exploratory result.
